# Opportunities and challenges of using generative AI to personalize educational assessment

**DOI:** 10.3389/frai.2024.1460651

**Published:** 2024-10-07

**Authors:** Burcu Arslan, Blair Lehman, Caitlin Tenison, Jesse R. Sparks, Alexis A. López, Lin Gu, Diego Zapata-Rivera

**Affiliations:** ETS Research Institute, Princeton, NJ, United States

**Keywords:** personalization, educational assessment, generative artificial intelligence, validity, reliability, fairness

## Abstract

In line with the positive effects of personalized learning, personalized assessments are expected to maximize learner motivation and engagement, allowing learners to show what they truly know and can do. Considering the advances in Generative Artificial Intelligence (GenAI), in this perspective article, we elaborate on the opportunities of integrating GenAI into personalized educational assessments to maximize learner engagement, performance, and access. We also draw attention to the challenges of integrating GenAI into personalized educational assessments regarding its potential risks to the assessment’s core values of validity, reliability, and fairness. Finally, we discuss possible solutions and future directions.

## 1 Introduction

Personalized learning has been shown to enhance learner motivation, engagement, and performance (Bernacki et al., 2021; Walkington, 2013; Walkington and Bernacki, 2018, 2019). Personalization can be delivered via humans (e.g., students or teachers), digital assessment systems (e.g., via a virtual agent embedded in a digital platform), or a combination (e.g., recommender systems). In educational assessment, standardization has been one of the most essential requirements for fair and valid measurement (Sireci, 2020). However, more recent discussions put the learners in front and expect that personalized assessments yield similar benefits to personalized learning (Bennett, 2023; Buzick et al., 2023; Sireci, 2020). The transition from standardized to more personalized assessment of learning (i.e., summative assessment) and assessment *for* learning (i.e., formative assessment) comes with inherent challenges in ensuring the validity, reliability, and fairness of more tailored, individualized assessments.

Artificial intelligence (AI) in education dates back more than four decades (see Holmes and Tuomi, 2022; Williamson and Eynon, 2020, for reviews). However, recent technological advancements and generative AI (GenAI) have broadened AI’s scale and potential applications in education due to its ability to create human-like text, being generic enough to be employed for different tasks, and real-time personalization capabilities. GenAI is a subcategory of AI designed to generate content, including images, videos, and text. Large language models (LLMs) are specifically trained on vast amounts of text data. When powered by LLMs, GenAI models can have a contextual understanding, enhanced memory, and create content based on natural language input (Hadi et al., 2023).

Recent research has increasingly focused on the integration of GenAI and LLMs in educational settings, examining their potential (e.g., Barany et al., 2024; Gökoğlu, 2024; Hu, 2023; Kasneci et al., 2023; Mazzullo et al., 2023; Nguyen et al., 2023; Olney, 2023; Pankiewicz and Baker, 2023; Pardos and Bhandari, 2024; Wang et al., 2022). Similarly, some studies focus on the application of GenAI and LLMs in educational assessment, exploring their impact and implications (e.g., Bulut et al., 2024; Hao et al., 2024; Jiang et al., 2024; von Davier, 2023; Swiecki et al., 2022).

Despite these advancements, to our knowledge, the potential opportunities and challenges of using GenAI to personalize educational assessment have not been explored. As we mentioned above, the shift from one-size-fits-all assessments to more culturally relevant and responsive approaches is becoming more critical, especially as stakeholders recognize the limitations of traditional assessments in responding to the needs of diverse populations. Thus, personalized educational assessments are increasingly viewed as a means to enhance learner engagement, performance, and access (Bennett, 2023; Buzick et al., 2023; Randall et al., 2022; Sireci, 2020).

Similar to the other application areas, advances in GenAI offer opportunities and challenges to personalized educational assessment (see Kirk et al., 2024, for benefits and risks of personalization in general with LLMs). GenAI can be integrated with the existing frameworks for including personalization, adaptation, or responsiveness in assessments, such as caring assessments (Lehman et al., 2024; Zapata-Rivera et al., 2020), socioculturally responsive assessments (Bennett, 2023), formative assessments (Bennett, 2011; Black and Wiliam, 2009), and intelligent tutoring systems (Corbett et al., 1997; Graesser et al., 2012). For example, in line with the caring assessment framework, GenAI may be leveraged to tailor content to the learner’s emotional, motivational, and cognitive state. Similarly, in line with socioculturally responsive assessments, GenAI may adapt assessment content to reflect diverse perspectives and contexts, considering the learner’s cultural background. Moreover, GenAI may enhance formative assessments and intelligent tutors by providing real-time, personalized feedback in a conversational style that helps learners improve continuously (e.g., Cheng et al., 2024). By leveraging these established frameworks, GenAI can offer robust personalized assessments that are not only effective but also responsive to the diverse needs of learners.

Integrating GenAI into the existing frameworks may play a crucial role in efficiently personalizing educational assessments by automatically generating images, videos, scenarios, and metadata and evaluating and scoring assessment items. Moreover, GenAI has the potential to generate or modify assessment items in real-time (Arslan, 2024), adapt to the learner’s responses, performance, interests, or cultural background, and provide personalized feedback and reporting dashboards. Additionally, GenAI can be used to create personalized conversations about the construct that can be used for assessment purposes to create assessment content at varying levels of language complexity or translate it into multiple languages. These potential uses of GenAI can help to achieve the previous efforts of enhancing the assessment experience through maximizing learner performance and engagement, activating existing *funds of knowledge* (González et al., 2005), and making assessments more relevant and accessible to learners, including neurodiverse and multilingual learners (Sireci, 2020).

However, using GenAI to personalize educational assessment also introduces significant challenges, such as ensuring fairness and maintaining validity and reliability. Research increasingly highlights the challenges and risks associated with GenAI, including issues such as bias, copyright infringement, the potential for harmful content, minimal control over its output, security concerns, and lack of interpretability and explainability (Bender et al., 2021; Kasneci et al., 2023). Table 1 shows the potential opportunities and challenges of using GenAI for personalized assessments, potential solutions, and future directions.

**Table 1:** Potential opportunities and challenges of using GenAI for personalized assessments, potential solutions, and future directions.

Opportunities	Challenges	Consequences	Potential solutions and future directions
Maximizing Engagement	Lack of control over the quality and content of the output (e.g., Bender et al., 2021; Jurenka et al., 2024). Hallucinations, potential bias and fairness issues (e.g., Jurenka et al., 2024; Hu, 2023; Jurenka et al., 2024; Jurenka et al., 2024; Jurenka et al., 2024; Jurenka et al., 2024; Jurenka et al., 2024; Ye et al., 2023; Jurenka et al., 2024). Lack of interpretability and explainability of the system’s underlying decision-making (see Jurenka et al., 2024 for a survey).	Jeopardizing assessment’s core values of: Validity. Reliability. Fairness (American Educational Research Association, American Psychological Association, National Council on Measurement in Education, Joint Committee on Standards for Educational and Psychological Testing (US), 2014; Jurenka et al., 2024).	Developing guidelines and standards for the ethical use of GenAI in personalized assessment (e.g., Hu, 2023; Jurenka et al., 2024). Aligning the purpose and goals of the assessment with how GenAI is being leveraged (Jurenka et al., 2024). Developing methodologies to evaluate the quality of the output of GenAI (e.g., human-in-the loop approaches; Jurenka et al., 2024; Jurenka et al., 2024; Jurenka et al., 2024). Working with practitioners and students to co-design solutions (Penuel, 2019). Identifying and implementing guardrails (Rai et al., 2024). Combining neuro-symbolic approaches and/or using computational cognitive architectures to create decision-making systems that leverage knowledge of human cognition (e.g., Jurenka et al., 2024; Jurenka et al., 2024).
On-the-fly context personalization (e.g., Arslan, 2024; Bennett, 2023; Bernacki and Walkington, 2018; Hidi and Renninger, 2006; Sireci, 2020; Walkington, 2013; Walkington and Bernacki, 2018, 2019).			
Conversational agents to enhance usability (Zapata-Rivera, 2012; Bull and Kay, 2016; Zapata-Rivera and Greer, 2002).			
Maximizing Performance			
Conversational agents to gather additional evidence, to provide just-in-time feedback (e.g., Kochmar et al., 2020; Ma et al., 2014; Mazzullo et al., 2023; Meyer et al., 2024; Matelsky et al., 2023; Pardos and Bhandari, 2024; Wang and Han, 2021), and to enhance dashboards for reporting (e.g., Forsyth et al., 2017; Xhakaj et al., 2017).			
Increasing Access			
Conversational agents, scaffolds, and language supports for neurodiverse and multilingual learners (e.g., Ali et al., 2020; López, 2023; Yang, 2024).			

## 2 Potential opportunities for applying GenAI in personalized assessments

GenAI may offer significant opportunities to enhance the personalization of assessments to maximize learner motivation and engagement, performance, and access.

### 2.1 Personalization for maximizing motivation and engagement

Increased motivation during test-taking leads to cognitive engagement, resulting in learners giving their best effort when answering assessment items (Finn, 2015; Wise and Kong, 2005; Wise, 2017). Cognitive engagement improves the likelihood that test scores will accurately represent what learners know and can do, as the interpretation of scores relies on the assumption that learners are trying their best (Finn, 2015; Wise, 2017). An effective way of maximizing engagement for learners with diverse interests and sociocultural is to personalize the context of assessment items (Bennett, 2023; Bernacki and Walkington, 2018; Sireci, 2020; Walkington and Bernacki, 2018). Context personalization can significantly enhance learner motivation and engagement by allowing learners to bring their cultural identity to the learning environment, leading to better learning outcomes (Walkington, 2013; Walkington and Bernacki, 2018, 2019).

LLMs have made it possible to personalize the context of assessment items *during* assessment based on each learner’s input about their interests embedded in their cultural identities, thus maximizing engagement through situational interest (see Hidi and Renninger, 2006) and has the potential to allow learners to show what they know and can do (Arslan, 2024). Unlike personalization approaches that leverage background variables (e.g., race/ethnicity) to create culturally relevant forms and assign each form to a group of learners based on their demographic information (e.g., Sinharay and Johnson, 2024), using LLMs offers real-time tailoring of content to individual interests and cultural background by providing learners agency and relevance that is often missing in standardized assessments. This approach acknowledges the diversity among learners, avoiding the pitfall of assuming homogeneity within groups (Arslan, 2024).

Integrating conversational virtual agents into assessment platforms is another way of making assessments more engaging and user-friendly. The virtual agents, powered by LLMs, can respond to queries in natural language, providing real-time, contextual support that assists both students and teachers during their interactions with the platform (Zapata-Rivera, 2012; Bull and Kay, 2016; Zapata-Rivera and Greer, 2002).

### 2.2 Personalization for maximizing performance

Unlike traditional summative assessments, personalized formative assessments can significantly enhance performance by providing feedback tailored to each learner’s needs (e.g., Kochmar et al., 2020; Ma et al., 2014; Mazzullo et al., 2023; Hu, 2023; Wang and Han, 2021). LLMs can generate hints and adaptive feedback during assessments at scale in an efficient way, helping learners understand their mistakes and learn from them in real-time (e.g., Meyer et al., 2024; Matelsky et al., 2023; Pardos and Bhandari, 2024) and facilitate adaptive conversations that guide learners through their thought processes (Hu, 2023; Forsyth et al., 2024; Zapata-Rivera et al., 2024).

LLMs can also enhance reporting by providing detailed, narrative insights for learners, teachers, and other interest holders. These insights can help interest holders understand assessment information more deeply and make informed decisions. For example, it can influence what teachers know about their student and their decision-making through dashboards (e.g., Forsyth et al., 2017; Xhakaj et al., 2017).

### 2.3 Personalization for increasing access

Personalized assessments can be crucial in increasing access for diverse learner populations, including neurodiverse and/or multilingual learners. LLMs offer various options for making assessments more linguistically responsive to the needs of multilingual learners (Yang, 2024). A significant way LLMs can enhance accessibility for multilingual learners is by providing support and scaffolds, such as translations to the learner’s preferred language, language simplification, glossaries, and read-aloud features. These tools allow multilingual learners—who comprise 10.6% of the student population in US public schools (National Center for Education Statistics, 2024a)—to utilize all available linguistic resources without compromising the construct being measured. These tools offer multilingual learners alternative ways to access and engage with assessment content, ensuring that language barriers do not block learners’ ability to fully demonstrate what they know and can do (Bennett, 2023; Sireci, 2020). In this context, LLMs are leveraged to provide enriched, inclusive means for all learners to access the assessment content and showcase their conceptual understanding using multiple modes of communication (e.g., linguistic, visual, aural, spatial, gestural) to reflect the diversity of needs and abilities in U.S. public schools (National Center for Education Statistics, 2024b). In essence, LLMs allow learners to use their entire linguistic repertoire, enabling them to express their KSAs through multiple forms of representation, including oral and written language and drawings (García and Wei, 2014; López, 2023). This approach, associated with translanguaging, supports providing multiple forms of expression, making assessments more inclusive and reflective of learners’ diverse backgrounds (Bennett, 2023).

Conversational virtual agents powered by LLMs can also be used to further support usability for neurodiverse and/or multilingual learners by enabling interactive, natural language-based supports with a choice of spoken and written communication in understanding and navigating the assessment platform, interpreting assessment items, and providing real-time, context-sensitive assistance. (e.g., see Ali et al., 2020). This potential application makes the platform more user-friendly, as discussed in the above section, and may ensure that all learners, regardless of their language proficiency, can fully participate in the assessment process.

## 3 Challenges, potential solutions, and future directions

Despite its potential, GenAI introduces significant challenges for personalized assessments. In this section, we first mention GenAI’s challenges in this context. Subsequently, we provide an overview of potential solutions to these challenges and future directions for research.

### 3.1 Challenges

Alongside research applying GenAI to new problems and domains, a growing body of work highlights the limitations and risks associated with its use. These discussions address potential biases, copywriting infringement, and the harmful content that can be introduced by large training datasets over which users have little control (Bender et al., 2021). Additionally, concerns about data privacy and security, particularly in educational contexts, are increasingly relevant when using these models (Kasneci et al., 2023). These general issues pose specific challenges when considering how GenAI can be used responsibly to support the design, administration, and reporting of personalized assessments while upholding the core values of validity, reliability, and fairness (see Johnson, 2024). Although these challenges may vary depending on the type and purpose of the assessment (e.g., formative vs. summative), we discuss several overarching challenges that are likely to shape the future development of personalized assessments using GenAI.

Personalizing assessments with GenAI offers benefits such as reducing construct-irrelevant variance indirectly by maximizing engagement (e.g., see Section 2.1) or directly by maximizing access (e.g., Section 2.3). However, without careful use, GenAI is just as likely to introduce new sources of construct-irrelevant variance. Approaches like Evidence-Centered Design (ECD; Mislevy et al., 2003) systematically align every aspect of the assessment process with theoretical and empirical evidence needed to support the claims made based on test scores. Part of the strength of this approach for generating valid assessments is the transparency at each step of the assessment development process and mapping decisions made to the intended interpretations and uses of the test. When using GenAI for on-the-fly content generation (e.g., see Section 2.1) or as a conversational virtual agent (e.g., see Sections 2.1 and 2.3), the lack of control over the output of GenAI makes it harder to ensure that the assessment content is measuring what we intend to measure (e.g., see Hong et al., 2024). With less control over the content, risks span from the introjection of inappropriate (e.g., see Greshake et al., 2023), non-sensical (Ye et al., 2023), incorrect (Hicks et al., 2024), or biased content and representations that these models have been known to exhibit (Cheung et al., 2024; Jiang et al., 2024; UNESCO, IRCAI, 2024; Schleifer et al., 2024; Zhou et al., 2024). Moreover, LLMs perform complex computations, complicating the interpretation of their decision-making processes. This ‘black-box’ nature of GenAI makes it harder to detect the sources of problematic output and to create explanations for interest holders (see Zhao et al., 2024 for a survey of the explainability of LLMs).

One of the cornerstone principles of standardized summative assessments is the consistency of test forms and the comparability of scores, which ensures the reliability and validity of scores across different test administrations (American Educational Research Association, American Psychological Association, National Council on Measurement in Education, Joint Committee on Standards for Educational and Psychological Testing (US), 2014). For example, in standardized summative assessments, on-the-fly personalization with GenAI, which generates uniquely tailored items during the assessment, may introduce construct-irrelevant variance into the measurement. This poses significant challenges to critical tenets of reliability and validity and complicates the currently established process for evaluating and documenting the reliability or precision of a given assessment. These challenges add a new dimension to ongoing discussions of the need for an expanded psychometric toolbox (such as computational psychometrics; von Davier et al., 2021), as well as more explicit guidance on valid score inferences when incorporating AI (Huggins-Manley et al., 2022) and personalization in assessments (Buzick et al., 2023).

Finally, developing and maintaining GenAI models specialized for personalized assessments involves numerous technical challenges. While prompt engineering is a popular method for adjusting a GenAI model’s behavior, its ability to alter the model’s actions is limited to what the model has already learned during pre-training (Bozkurt and Sharma, 2023; Jurenka et al., 2024). Alternative approaches like fine-tuning are much more expensive, requiring both quality data and expertise (e.g., Chevalier et al., 2024). Lastly, concerns regarding the hosting and management of GenAI models highlight critical data privacy and security issues. These are especially pertinent in educational contexts, where the sensitivity of learner data requires stringent security measures and ethical considerations (see Johnson, 2024).

### 3.2 Potential solutions and future directions

There are several key areas for future research to understand better how GenAI can be leveraged for personalized assessments. The first area is identifying, developing, and distributing guidelines and standards for the ethical use of GenAI in personalized assessments. A set of guidelines and standards helps guide future research and development and facilitates clear expectations for interest holders. Several emerging efforts exist to establish responsible AI standards in educational assessment (Burstein, 2023; Johnson, 2024). However, continued work is needed to establish guidelines and standards that encompass the full potential uses of GenAI in assessment design and development (e.g., content development to be evaluated by humans vs. on-the-fly personalization). To this end, as we briefly mentioned in the Introduction, existing frameworks for personalization, adaptation, and responsiveness in assessments may help identify these potential uses and important use cases.

The second area for future research is identifying how to best leverage GenAI in different testing contexts. As is typically the case in education, there is unlikely to be a one-size-fits-all solution for leveraging GenAI for personalized assessments (Bennett, 2023). For example, on-the-fly item generation may not be appropriate for a summative, high-stakes assessment in which there are high demands for score comparability. However, it may be appropriate for a formative, low-stakes assessment in a classroom context. Moreover, when additional approaches are taken to mitigate the inherent challenges of GenAI (e.g., nonfactual information and bias), it may be appropriate to leverage it to provide learners with conversational support during the assessment. Thus, it is essential to align the purpose and goals of the assessment with how GenAI is being leveraged and to develop methodologies to evaluate the quality of the output of GenAI before operationalizing the personalized assessments (e.g., see Zapata-Rivera et al., 2024 for leveraging ECD). It will be critical to regularly evaluate the impact of using GenAI-developed content for assessments on the perceptions of various interest holders when applied in different manners to different testing contexts. (e.g., teachers, learners, policymakers). It will also be essential to leverage GenAI to address the current needs of practitioners (e.g., teachers, assessment developers) and learners to improve the experience of developing, administering, and completing assessments. For example, teachers may struggle to provide all aspects of students’ Individualized Education Programs (IEPs) during an assessment due to tools without the appropriate nuance and/or resource limitations, such as one teacher in a class of 30 students (Lehman et al., submitted).^1^ Researchers can work with practitioners and students to co-design solutions to these real-world problems that utilize GenAI (Penuel, 2019).

When establishing how best to leverage GenAI in different testing contexts, a third area of research is needed to identify the guardrails that must be implemented to address some of the abovementioned challenges. While it may be tempting to let GenAI run free to maximize its potential benefits fully, key guardrails can be implemented to limit unintended negative consequences and maintain rigorous, appropriate content for personalized assessments. For example, implementing a ‘human-in-the-loop’ approach allows for human inspection and evaluation before GenAI-generated content is presented to learners (Amirizaniani et al., 2024; Drori and Te'eni, 2024; Park, 2024). However, this type of human review can limit some potential uses of GenAI, such as on-the-fly personalization. Moreover, rigorous research is essential to narrow the decision space for GenAI and mitigate the ‘black-box’ nature of LLMs. This can be achieved by integrating neuro-symbolic approaches or using computational cognitive architectures to develop decision-making systems that leverage an understanding of human cognition (e.g., Sumers et al., 2023; Sun, 2024). Additionally, combining these approaches with insights from key interest holders—such as teachers, students, and assessment developers—can help identify effective ways to utilize GenAI while minimizing unintended negative consequences.

The previous areas for future research have focused on the content generation process via GenAI. However, there is also a need for rigorous research to evaluate the personalized assessments that are developed with GenAI. This research will need to evaluate the quality of the content developed with or by GenAI and how the use of GenAI impacts the broader uptake of personalized assessments. When appropriate, it will be necessary to evaluate the utility of GenAI content within the current assessment development process. For example, it will be necessary to document if GenAI content results in more efficient content development processes that still maintain high levels of quality (e.g., see Park, 2024). Another area for future research is how GenAI could be leveraged to support response scoring, which could support personalized assessment and more efficient reporting (e.g., Section 2.2.).

Lastly, the full potential of utilizing GenAI for personalized assessments can only be realized if interest holders (e.g., teachers, learners, curriculum experts, and policymakers) view those assessments as valid, reliable, and fair, thus trustworthy and helpful in supporting learning.

## 4 Conclusion

Overall, there is a significant opportunity to enhance the deployment and effectiveness of personalized assessments, which could offer learners more relevant test materials, leading to greater engagement, improved performance, and broader access. This, in turn, has the potential to produce more valid test outcomes. However, while the potential of GenAI to create more valuable assessments is promising, it is crucial to proceed with caution. The field must continue to explore how GenAI can be effectively harnessed, but this exploration should be grounded in a rigorous evaluation of its utility.

As we move forward, it is essential not to abandon the potential for future advancements in assessments in favor of holding onto outdated development and evaluation processes (Huggins-Manley et al., 2022; Sireci, 2020). While embracing the possibilities offered by AI, we must ensure that these new tools are evaluated against criteria that recognize the affordances of both current and future technologies. However, this should never come at the expense of the core values of assessments—validity, reliability, fairness, and alignment with valued educational goals. By balancing innovation with caution, we can strive to create assessments that are both cutting-edge and trustworthy.

## Footnotes

^1^Lehman, B., Gooch, R., Tenison, C., & Sparks, J. R. (submitted). The role of teachers in digital personalized assessments. Paper submitted to the annual meeting of the American Educational Research Association. Denver, CO.
